# Bilateral Corneal Dystrophy Revealing Mucolipidosis Type IV: A Case Report

**DOI:** 10.7759/cureus.112877

**Published:** 2026-07-17

**Authors:** Boutaina Bousellam, Hibat Allah Eddaoui, Aniss Regragui, Nabiha Benchekroun, Mohamed Belmekki

**Affiliations:** 1 Ophthalmology, Abulcasis International University of Health Sciences, Cheikh Zaid Hospital, Rabat, MAR

**Keywords:** as-oct findings, corneal dystrophy, genetic counseling, lysosomal disease, mcoln1, mucolipidosis type iv, pediatric ophthalmology

## Abstract

A nine-year-old boy, the only child of non-consanguineous parents, presented with progressive bilateral visual impairment and corneal clouding since birth. Ocular examination showed bilateral epithelial-stromal corneal dystrophy associated with photophobia, blepharospasm, alternating esotropia, and nystagmus. Visual acuity was limited to light perception in both eyes, with normal intraocular pressure. Anterior segment optical coherence tomography (OCT) demonstrated diffuse epithelial-stromal thickening with hyperreflective anterior stroma. Flash electroretinography (ERG) was normal, while flash visual evoked potentials (VEP) revealed bilateral optic neuropathy. Systemic examination noted severe psychomotor impairment, hypotonia, and facial dysmorphism. Whole-exome sequencing identified a homozygous nonsense mutation in MCOLN1 (c.169C>T p.Arg57*), confirming mucolipidosis type IV. Mucolipidosis type IV is a rare autosomal-recessive lysosomal storage disorder combining ocular and neurological manifestations. In children with congenital corneal opacity and developmental delay, metabolic and genetic evaluation should be systematically pursued. Corneal transplantation is not recommended because of recurrence risk; management is supportive and multidisciplinary.

## Introduction

Mucolipidosis type IV (MLIV) is an extremely rare autosomal recessive lysosomal storage disorder caused by biallelic pathogenic variants in the MCOLN1 gene (19p13.2), which encodes mucolipin-1 (TRPML1), a lysosomal cation channel involved in endolysosomal trafficking and intracellular ion homeostasis [1,2]. Unlike most lysosomal storage disorders, lysosomal hydrolase activities remain normal despite progressive intracellular accumulation of storage material [3,4]. MLIV is rare worldwide but occurs more frequently among individuals of Ashkenazi Jewish ancestry due to two founder mutations [3,5].

The disease typically presents during infancy with developmental delay, hypotonia, psychomotor impairment, and progressive loss of visual acuity. Bilateral corneal clouding is often one of the earliest clinical manifestations and may precede the full neurological phenotype, making ophthalmologists crucial for early recognition. Because congenital bilateral corneal opacity has a broad differential diagnosis, including congenital hereditary corneal dystrophies, mucopolysaccharidoses, and cystinosis, the diagnosis of MLIV remains challenging, particularly in non-Jewish populations [5-7]. We report the case of a Moroccan child with congenital bilateral corneal clouding due to a rare MCOLN1 splice-site variant, highlighting the importance of considering MLIV in the differential diagnosis of congenital bilateral corneal opacity and the role of molecular genetic testing in establishing an early diagnosis.

This case deserves attention for several reasons. First, MLIV has been reported predominantly in patients of Ashkenazi Jewish ancestry, and diagnostic suspicion may be delayed in populations where the disease is not classically expected; this Moroccan case therefore expands the recognized ethnic and geographic spectrum of MLIV. Second, the MCOLN1 variant identified in our patient has rarely been reported in the clinical literature, contributing additional genotype-phenotype data. Third, this case highlights the potential diagnostic value of anterior segment optical coherence tomography (OCT) as a readily available, non-invasive imaging modality that may raise suspicion for a lysosomal storage disorder before systemic or genetic confirmation, particularly in settings where access to molecular testing is limited. Finally, early recognition of MLIV has important clinical implications, as it may prevent unnecessary corneal transplantation while enabling timely genetic counselling for affected families.

## Case presentation

Patient history

A nine-year-old boy, the only child of non-consanguineous parents, presented with bilateral visual loss and congenital corneal opacities. Family history was notable for recurrent first-trimester miscarriages in both his mother (four) and maternal grandmother (three). The pregnancy and delivery were unremarkable.

From the age of six months, the child had experienced generalized epileptic seizures. He also exhibited psychomotor delay and axial hypotonia. On general examination, facial dysmorphism was observed, including midface hypoplasia, flat nasal bridge, and relative microcephaly; other systemic findings were unremarkable.

Ophthalmic examination

**Figure 1:**
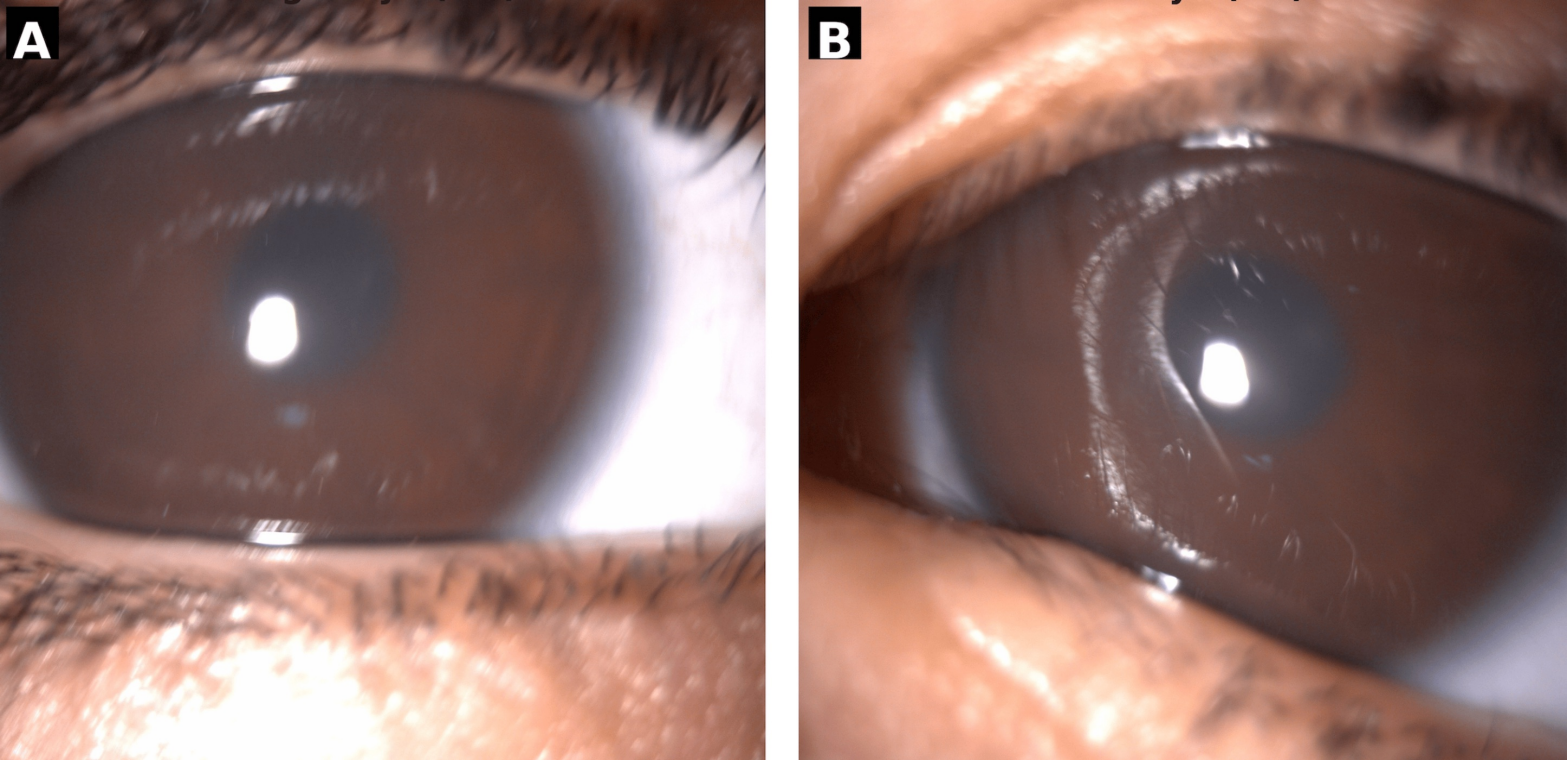
Slit-lamp biomicroscopy demonstrating bilateral epithelial-stromal corneal dystrophy: (A) right eye and (B) left eye

Best-corrected visual acuity was limited to light perception in both eyes. Intraocular pressure was normal bilaterally. Slit-lamp biomicroscopy demonstrated bilateral epithelial-stromal corneal dystrophy with a clear lens and deep quiet anterior chamber. Ocular motility assessment revealed alternating esotropia and manifest latent nystagmus. Fundus visualization was not possible due to the severity of corneal opacification (Figure 1).

Investigations

**Figure 2:**
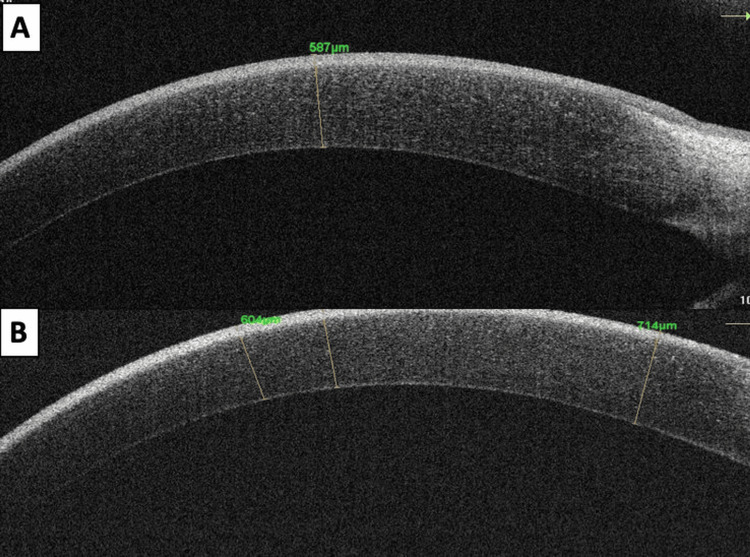
Anterior segment optical coherence tomography showing epithelial-stromal hyperreflectivity: (A) right eye and (B) left eye

Anterior segment OCT demonstrated diffuse epithelial-stromal hyperreflectivity with anterior stromal thickening; corneal pachymetry measured 587 μm in the right eye and 604 μm in the left eye (Figure 2).

**Figure 3:**
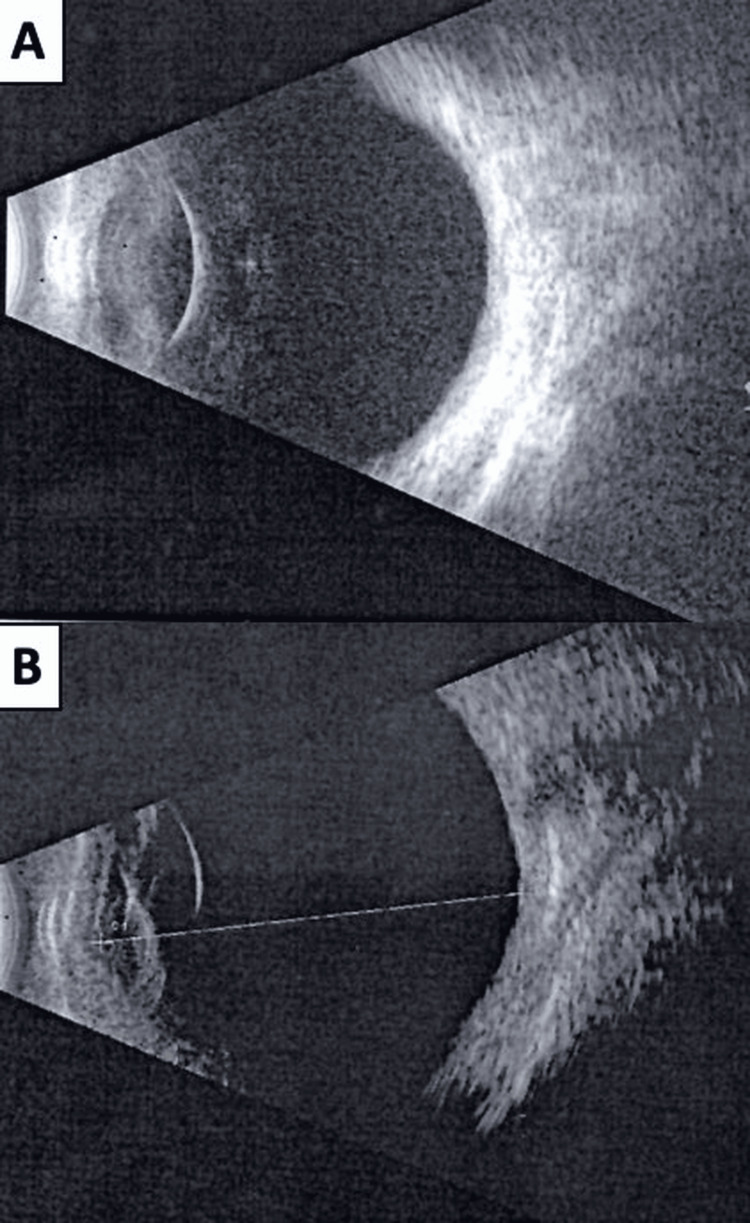
Ocular ultrasound (B-scan) showing a normal posterior segment: (A) right eye and (B) left eye

B-scan ultrasonography showed a normal posterior segment with a flat retina bilaterally (Figure 3).

Electrophysiological testing was performed: flash electroretinography (ERG) was within normal limits, whereas flash visual evoked potentials (VEP) revealed markedly reduced bilateral P100 amplitudes, consistent with bilateral optic neuropathy.

Genetic analysis

**Figure 4:**
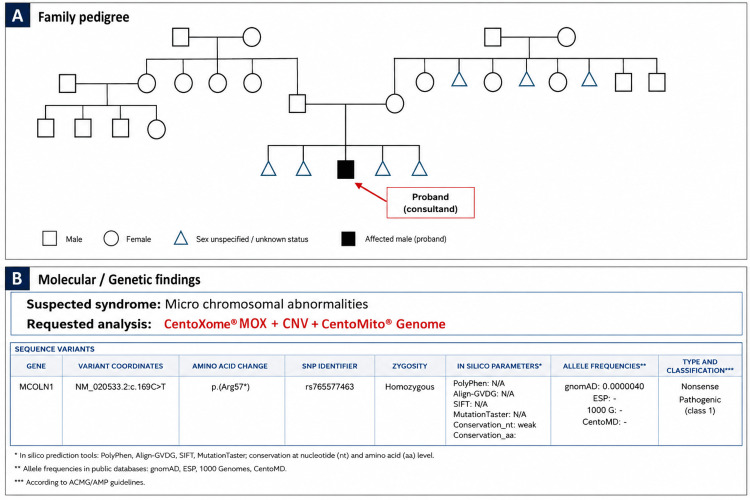
Pedigree and molecular genetic findings (A) Family pedigree showing the affected proband (black square) born to unaffected parents. (B) Molecular genetic analysis identifying a homozygous pathogenic MCOLN1 variant, c.169C>T (p.Arg57*), consistent with the diagnosis of mucolipidosis type IV. The figure was created using Microsoft PowerPoint (Microsoft Corporation, Redmond, Washington, United States).

Whole-exome sequencing revealed a homozygous nonsense variant in MCOLN1 (c.169C>T, p.Arg57*), introducing a premature stop codon in exon 2 (of 14) and resulting in loss of normal protein function (Figure 4). The variant is listed in the ClinVar database. However, to the best of our knowledge, published clinical reports describing patients harboring this specific variant remain limited, highlighting the rarity of this molecular finding.

Diagnosis

Based on the clinical presentation, namely, congenital bilateral corneal dystrophy, epilepsy, psychomotor retardation, axial hypotonia, facial dysmorphism, and the identified homozygous loss-of-function variant in MCOLN1, a diagnosis of MLIV was established.

Management

The patient was referred for multidisciplinary follow-up involving neurology, ophthalmology, and clinical genetics. Ophthalmic management was directed at visual rehabilitation and supportive care. Genetic counselling was offered to the family given the autosomal recessive inheritance pattern of the condition.

## Discussion

MLIV is a rare autosomal recessive neurodegenerative lysosomal disorder caused by pathogenic variants in the MCOLN1 gene, resulting in defective lysosomal ion transport and vesicular trafficking with progressive intracellular accumulation of undegraded substrates. Unlike most lysosomal storage disorders, lysosomal hydrolase activities remain normal, which represents a distinctive biochemical hallmark of the disease [1-3].

More than 80% of reported patients are of Ashkenazi Jewish origin because of two founder mutations. Nevertheless, MLIV has been increasingly recognized in other ethnic groups, with several reports describing non-Jewish patients carrying diverse pathogenic MCOLN1 variants and exhibiting both classical and milder phenotypes [4,5,8].

Ocular manifestations are among the earliest and most consistent clinical features of MLIV and often represent the first diagnostic clue. Progressive bilateral corneal clouding, photophobia, strabismus, nystagmus, optic nerve pallor, and retinal degeneration have all been well described [6,7]. Anterior segment OCT frequently demonstrates epithelial and anterior stromal hyperreflectivity corresponding to intracellular storage material, providing a valuable non-invasive diagnostic tool [5,7]. Neurological manifestations, including axial hypotonia, developmental delay, cognitive impairment, and epilepsy, usually become evident during infancy and progressively worsen over time [3,5].

The differential diagnosis includes mucopolysaccharidoses, cystinosis, and congenital hereditary corneal dystrophies. Definitive diagnosis relies on the identification of biallelic pathogenic MCOLN1 variants by molecular genetic testing [1,2,7]. To date, no curative treatment is available, and management remains supportive, focusing on visual rehabilitation, ocular surface protection, neurological care, and genetic counselling. Corneal transplantation is generally discouraged because storage material typically recurs within the donor graft [6,7].

The MCOLN1 gene encodes TRPML1, a lysosomal transient receptor potential cation channel that plays a fundamental role in endolysosomal trafficking, membrane fusion, autophagy, and intracellular ion homeostasis [9]. Pathogenic variants impair lysosomal function, leading to progressive storage material accumulation and the multisystem manifestations characteristic of MLIV [8,9].

The c.169C>T, p.Arg57* variant identified in our patient introduces a premature stop codon in exon 2 of MCOLN1, resulting in a truncated, non-functional mucolipin-1 protein and, presumably, the degradation of the aberrant transcript by nonsense-mediated decay. This loss-of-function mechanism is predicted to result in markedly reduced or absent TRPML1 function. Although this variant is listed in ClinVar, reports describing affected individuals remain limited [10].

Previously reported pathogenic MCOLN1 variants include nonsense, frameshift, splice-site, and large deletion mutations, all leading to loss of protein function [8,9]. Consistent with these established pathogenic mechanisms, the c.169C>T, p.Arg57* variant is expected to produce a loss-of-function effect in keeping with the autosomal recessive inheritance pattern and the clinical phenotype observed in our patient [9,10]. The identification of this rare nonsense variant further expands the molecular spectrum of MCOLN1-associated disease and underscores the importance of genetic testing in children presenting with unexplained congenital corneal clouding associated with neurodevelopmental abnormalities.

Our patient's presentation, that is, bilateral congenital corneal opacity, nystagmus, strabismus, epilepsy, axial hypotonia, and psychomotor regression, closely mirrors the classical MLIV phenotype reported in the literature [3-7]. As in previously published cases, ocular manifestations were the earliest clinical findings and prompted the diagnostic work-up, emphasizing their importance in recognizing the disease before the neurological phenotype becomes fully apparent.

From a practical ophthalmic perspective, this case highlights two important clinical messages. First, MLIV should be considered in any child presenting with bilateral congenital corneal clouding associated with hypotonia or developmental delay, even in the absence of skeletal, visceral, or dysmorphic features suggestive of other lysosomal storage disorders and irrespective of ethnic origin or parental consanguinity [5,8,9]. In our patient, anterior segment OCT objectively localized hyperreflective deposits to the corneal epithelium and anterior stroma, a characteristic pattern that may help distinguish MLIV from mucopolysaccharidoses, cystinosis, and congenital hereditary corneal dystrophies while providing a useful non-invasive tool for longitudinal follow-up [5,7].

Second, although our patient's electroretinogram remained normal, retinal dysfunction may develop later during disease progression. Consequently, electrophysiological testing should be repeated periodically rather than interpreted as reassuring after a single normal examination [7]. Genetic confirmation remains essential not only for establishing the diagnosis but also for prognostic counselling and reproductive planning, particularly in families with a history suggestive of inherited disease.

Finally, as reported in previous studies, corneal transplantation was not considered because recurrence of storage material within the graft has consistently been described, limiting long-term visual benefit. Therefore, visual rehabilitation, low-vision support, and multidisciplinary follow-up remain the cornerstones of ophthalmic management [6,7].

## Conclusions

This case highlights the importance of considering metabolic and genetic disorders in children presenting with bilateral corneal opacity and neurodevelopmental delay, even outside populations classically associated with MLIV. In our patient, anterior segment OCT provided an early, non-invasive clue that raised diagnostic suspicion before genetic confirmation, supporting its value in the evaluation of congenital corneal clouding associated with hypotonia, developmental delay, or a family history suggestive of an autosomal recessive disorder. Identification of the pathogenic MCOLN1 variant confirmed the diagnosis, enabled appropriate genetic counselling in the context of recurrent miscarriages, and avoided an unnecessary corneal transplantation that was unlikely to provide sustained visual benefit. This report also expands the limited literature on MLIV in non-Jewish populations and underscores the pivotal role of ophthalmologists in recognizing this rare disorder.
